# Advances in Nutritional Manipulation of Rumen Fermentation

**DOI:** 10.3390/ani15060833

**Published:** 2025-03-14

**Authors:** Lizhi Wang

**Affiliations:** Institute of Animal Nutrition, Sichuan Agricultural University, Chengdu 611130, China; wanglizhi08@aliyun.com

## 1. Introduction

Rumen, a complex and dynamic ecosystem, plays a pivotal role in the digestion and nutrient utilization of ruminant animals. The complex microbial communities and fermentation processes in rumen have a profound impact on feed utilization efficiency, animal performance, and environmental sustainability. In this regard, this Special Issue, ‘Advances in Nutritional Manipulation of Rumen Fermentation’ (*Animals*, 2025), brings together the latest research in this field and provides an important scientific basis and practical guidance for optimizing ruminant production.

Rumen fermentation is a critical process in ruminant nutrition, as it determines the efficiency of feed utilization and the production of volatile fatty acids (VFAs), which are essential for energy metabolism. However, aberrant rumen fermentation can lead to various health issues, including rumen acidosis and rumen bloat, which can have severe consequences for animal welfare and productivity. Nutritional manipulation offers a promising means of addressing these challenges by regulating rumen fermentation, improving feed efficiency, and reducing the incidence of rumen disorders.

## 2. Methodological Breakthroughs

This Special Issue presents examples of how advanced approaches have deepened our understanding of rumen fermentation. The following two key approaches stand out.

### 2.1. In Vitro Fermentation Models

This Special Issue highlights the utility of in vitro systems for hypothesis testing without ethical or logistical constraints. Lukbun et al. (2024) utilized a dual-flow continuous culture system to simulate rumen conditions, demonstrating that cassava-based diets supplemented with Enterococcus gallinarum KKU-BC15 reduced cyanide toxicity by 98% while increasing propionate concentration by 8.97% [1]. This model not only accelerates the screening of feed additives, but also mitigates risks associated with in vivo trials, such as acute acidosis or cyanide poisoning. Such systems are pivotal for translating lab discoveries into practical farm solutions.

### 2.2. Application of Meta-Analysis Methods

Meta-analysis is now widely used in various fields. In the present Special Issue, the study by Yanza et al. (2024) presents the first systematic meta-analysis of the effects of different sources of saponin extracts in ruminants, revealing their potential role in ruminant nutrition and environmental management [2]. Their database collection of 26 articles, containing 66 in vivo studies, was analyzed using mixed models with SAS software, and their results show that saponin extracts can significantly affect production performance, rumen fermentation, nitrogen utilization, and blood metabolites in ruminants, but that the effects of saponin extracts varied among different sources and levels.

## 3. Addressing Persistent Challenges in Ruminant Nutrition

### 3.1. Methane Mitigation

Rumen fermentation accounts for ~30% of anthropogenic methane emissions, a critical target for climate action. This Special Issue contains articles that explore the reduction in methane emissions using additives. Li et al. (2024) investigated the methane mitigation effects and the mechanisms of *Sargassum mcclurei* using in vitro rumen fermentation [3]. The study found that freeze-dried *S. mcclurei* at a 2% supplementation level significantly reduced methane emissions and enhanced crude protein degradability. This research highlights the potential use of *S. mcclurei* as a feed additive to reduce methane emissions and improve rumen fermentation.

### 3.2. Protein and Energy Levels

Optimizing the ratio of energy to protein in diets can effectively improve feed costs and increase feed utilization efficiency. Jo et al. (2024) explored the effects of different incubation temperatures on ruminal fermentation and rumen microorganisms, aiming to determine the appropriate protein and energy levels for the enhancement of microbial protein synthesis [4]. The study found that higher incubation temperatures increased NH3-N and total volatile fatty acids (TVFAs), but decreased liquid-associated bacterial (LAB) amounts. The findings suggest that dietary adjustments can significantly impact rumen fermentation and microbial activity, providing insights into metabolic adaptation under different ruminal temperatures.

### 3.3. Development and Utilization of Feed Resources

The rational development and utilization of feed resources is an effective measure to effectively reduce feed costs and improve economic efficiency. The study by Lukbun et al. (2024) investigated the effects that adding cyanide-utilizing bacteria (CUB) to cassava diets had on rumen fermentation, gas production, and cyanide concentrations [1]. The results show that supplementing cassava diets with CUB, particularly at high cyanide levels, significantly improved cyanide degradation, gas production, and in vitro digestibility. This study highlights the potential for CUB to mitigate cyanide toxicity and enhance feed quality, which is particularly relevant for regions where cassava is a primary feed source.

### 3.4. Innovations and Applications of Nutritional Strategies

The research in the Special Issue also explores the practical effects of a variety of nutritional regulation strategies. For example, the addition of specific feed additives (e.g., saponins, active dry yeast, and plant extracts) significantly improve rumen fermentation patterns, reduce methane emissions, and lower the risk of rumen acidosis and bloat. Liu et al. (2024) assessed the impact of active dry yeast (ADY) on nutrient digestibility and rumen fermentation using both in vitro and in vivo experiments [5]. Their results indicate that ADY supplementation improves nutrient digestibility and rumen fermentation, with the specific effects varying depending on the type of ADY used. This study underscores the potential for ADY to be used as a feed additive to enhance lamb growth and optimize rumen fermentation.

## 4. Future Research Directions

The studies in this Special Issue lay the foundation for future research in the field of rumen fermentation. For instance, the potential for cyanide-utilizing bacteria (CUB) to mitigate cyanide toxicity in cassava diets warrants further investigation to explore their applications in different ruminant species and feeding conditions. Similarly, the effects of different yeast strains and supplementation levels on rumen fermentation and animal performance need to be explored in more depth to optimize their use in practical feeding strategies.

The findings in regard to methane mitigation using *Sargassum mcclurei* open up new avenues for research into the use of seaweed and other natural products as feed additives to reduce greenhouse gas emissions from ruminants. Additionally, attempts to explore the use of dietary adjustments to enhance microbial protein synthesis and improve feed efficiency under different environmental conditions provides a basis for developing tailored nutritional strategies for ruminants.

## 5. Conclusions

In summary, this Special Issue, ‘Advances in Nutritional Manipulation of Rumen Fermentation’, presents a comprehensive collection of research articles that explore the latest advancements in the field of ruminant nutrition. The studies highlight the potential for various nutritional strategies to optimize rumen fermentation, enhance feed efficiency, and address common rumen disorders. The findings provide valuable insights for researchers, farmers, and industry stakeholders, offering practical solutions to improve animal productivity and sustainability.

By showcasing methodological breakthroughs and addressing persistent challenges, this Special Issue contributes significantly to the current academic discourse on rumen fermentation. The research presented here not only advances our understanding of the complex interactions between nutrition and rumen microbiota, but also provides a roadmap for future research and applications in the field of ruminant nutrition.
